# Clinical Clues to Differentiate between Dermatophyte Onychomycosis (DP-OM) and Dermatophytoma-Like Traumatic Onychodystrophy (DP-TO)

**DOI:** 10.1155/2022/8519376

**Published:** 2022-09-09

**Authors:** Sumanas Bunyaratavej, Penvadee Pattanaprichakul, Panitta Sitthinamsuwan, Bawonpak Pongkittilar, Suthasanee Prasertsook, Supisara Wongdama, Chadakan Yan, Charussri Leeyaphan

**Affiliations:** ^1^Department of Dermatology, Faculty of Medicine Siriraj Hospital, Mahidol University, 2 Wanglang Road, Bangkok Noi, Bangkok 10700, Thailand; ^2^Department of Pathology, Faculty of Medicine Siriraj Hospital, Mahidol University, 2 Wanglang Road, Bangkok Noi, Bangkok 10700, Thailand

## Abstract

**Background:**

Dermatophytoma is a recalcitrant condition of onychomycosis (OM). It presents as a white- or yellow-colored fungal mass that appears linear/triangular or round on a nail plate. Traumatic onychodystrophy (TO) can present with dermatophytoma-like lesions. Typically, OM and TO are not clinically distinguishable. Mycological testing is the gold standard for differentiating these disorders.

**Objectives:**

This study is aimed at differentiating between the clinical and dermoscopic factors related to dermatophytoma onychomycosis (DP-OM) and dermatophytoma-like traumatic onychodystrophy (DP-TO).

**Methods:**

A retrospective study was conducted of patients with dermatophytoma-like nail lesions who visited the Siriraj Nail Clinic between January 2010 and July 2020. The diagnosis of DP-OM was made by direct microscopy, fungal cultures, and histopathology of nail clippings.

**Results:**

A total of 36 nails were included in the study. Thirteen nails were DP-OM, and 23 nails were DP-TO. The demographic data and risk factors for the 2 groups were not significantly different. Dermatophytoma lesions were found on the lateral side of nails in 12 cases of DP-OM (92.3%) and 11 cases of DP-TO (47.8%; *P* = 0.008). DP-OM was associated with longitudinal striae adjacent to dermatophytoma (69.2% vs. 30.4%; *P* = 0.024), sulfur-nugget-like subungual debris (23.1% vs. 0%; *P* = 0.040), and scale on the ipsilateral foot (69.2% vs. 8.7%; *P* < 0.001). DP-TO was associated with a homogenous, whitish discoloration (47.8% vs. 7.7%; *P* = 0.014) and a sharp edge of the onycholytic area (43.5% vs. 0%; *P* = 0.005).

**Conclusions:**

The lateral location of dermatophytoma, adjacent striae, sulfur-nugget-like debris, and scale on the ipsilateral foot were significantly associated with DP-OM. Dermoscopic examination (dorsal and hyponychium views) and foot examination are beneficial for distinguishing between DP-OM and DP-TO.

## 1. Introduction

Dermatophytoma, a form of distal-lateral subungual onychomycosis (OM), is clinically described as a white- or yellow-colored linear/triangular or round shape on a nail plate. It has been reported to be recalcitrant to topical and oral treatments. This resistance is suspected to be due to biofilm produced by fungus within the hyperkeratotic mass [1]. Microscopically, dermatophytoma onychomycosis (DP-OM) has numerous fungal hyphae and large spores that form a fungal mass [2]. A previous study has found DP-OM in 9% of patients with OM and was recognized as a poor prognostic factor for cure after oral treatment [3]. Dermatophyte and non-dermatophyte species can form DP-OM [2, 4]. Recognition of DP-OM is crucial because of the need for mechanical manipulation. However, this condition is underrecognized by physicians [5].

Traumatic onychodystrophy (TO) is an abnormal nail change due to repetitive exogenous traumatic injury [6]. The abnormal characteristics frequently observed are Beau's lines, onycholysis, and nail color change [7, 8]. The appearance of TO is sometimes similar to that of dermatophytoma, but investigations for fungal infection prove to be negative [9].

A laboratory investigation is necessary to differentiate between DP-OM and dermatophytoma-like traumatic onychodystrophy (DP-TO). Microscopic examination and histopathology of nail clippings show more sensitivity than culture [10, 11]. Nevertheless, a fungal culture is still the gold standard diagnostic test because of its ability to identify causative organisms [12]. Unfortunately, microscopic and histopathological examinations and cultures have limited sensitivity and specificity. Moreover, some investigations require several days to produce results. In addition to laboratory investigations, many studies have reported using dermoscopy to help discriminate between OM and TO [6, 13–16]. However, there are limited reports on discerning between DP-OM and DP-TO through dermoscopic examination. The current investigation aimed to establish the distinguishing risk factors, clinical and dermoscopic features of DP-OM and DP-TO, and their histopathological findings. The distinct features between DP-OM and DP-TO will facilitate the process of diagnosis, especially in area where laboratory investigations are limited.

## 2. Materials and Methods

The Siriraj Institutional Review Board approved the study design (SI713/2020). A retrospective study was conducted on patients with dermatophytoma-like nail lesions who underwent microscopic examination, fungal culture, and nail clipping for histopathology at the Nail Clinic, Department of Dermatology, Siriraj Hospital, between January 2010 and July 2020.

Records of the patients' medical histories (demographic data, comorbidities, risk factors associated with OM, and history of trauma) and their foot and nail examinations were reviewed. Two dermatologist experts in nail diseases evaluated photographs of the patients' feet and dermoscopic photographs of their nails' dorsal and hyponychium views. The dermatologists were blinded to the patients' mycological results.

DP-OM was defined by the presence of at least one of the following: (1) microscopy with 20% potassium hydroxide that was positive for septate hyphae, (2) a positive culture for molds, and (3) a positive histology for hyphae invading the nail plate.

### 2.1. Statistical Analysis

Analyses were calculated using PASW Statistics for Windows, version 18 (SPSS Inc., Chicago, IL, USA). Statistical tests were 2-sided, and “significance” was defined as probability (*P*) values <0.05. Data are shown as the mean with standard deviation for continuous data with a normal distribution and the median with interquartile range for continuous data with a non-normal distribution. Categorical data are presented as numbers and percentages. For comparisons of continuous data, Student's *t*-test was used for normally distributed data, and the Mann–Whitney *U* test was used for non-normally distributed data. Categorical data were compared using the chi^2^ test or Fisher's exact test.

## 3. Results

Thirty-three patients were enrolled. Thirteen patients had DP-OM based on positive mycology. Causative organisms were dermatophytes (38.5%: *Trichophyton rubrum*, 23.1%, and *T. mentagrophytes*, 15.4%) and non-dermatophytes (38.5%: *Neoscytalidium* spp., 15.4%; *Fusarium* spp., 15.4%; and *Aspergillus* spp., 7.7%). Three patients with positive potassium hydroxide or nail histology had negative fungal cultures. In contrast, 20 patients were classified as DP-TO. Women represented 53.8% of the patients in the DP-OM group and 75% of the DP-TO group. The mean ages were 60.6 ± 15.7 years for the DP-OM group and 61.4 ± 8.2 years for the DP-TO group. There were no statistically significant differences in the comorbidities of the 2 groups (hypertension, dyslipidemia, diabetes mellitus, chronic kidney disease, and chronic vascular insufficiency). In addition, there were no significant associations between either the demographic data or the predisposing factors of the DP-OM and DP-TO groups.

The nail and foot findings are detailed in Table 1. A total of 36 nails were included in the analysis (13 DP-OM and 23 DP-TO). Three DP-TO patients had 2 abnormal nails, each of which was DP-TO. Regarding the locations of the dermatophytoma lesions, the central location was statistically associated with DP-TO, whereas the lateral location was associated with DP-OM (*P* = 0.008). As for plantar scale, suspected tinea pedis was more significantly found in patients with DP-OM. Among 14 patients with fungal tests from the foot and ipsilateral tinea pedis, positive fungal results were found only in the DP-OM group (90.0% vs. 0%; *P* = 0.002). As to the dermoscopic findings, DP-TO was significantly associated with onycholytic lesions with homogenous, whitish discoloration and sharp edges. On the other hand, DP-OM was significantly associated with yellow, clumping, subungual debris (having a “sulfur-nugget-like” appearance), and longitudinal striae adjacent to dermatophytoma (Table 2).

Nail histopathology results for the dermatophytoma lesions are summarized in Table 3. There were no significant differences in the degrees and locations of parakeratosis in the 2 groups. However, 2 cases of DP-OM showed parakeratosis on the lower half of the nail plate. In contrast, 2 cases of DP-TO had parakeratosis solely on the upper half of the nail plate. The remaining cases had diffuse parakeratosis throughout the nail plate. Among patients with DP-OM, clumping fungal hyphae were found in 3 cases (23.1%) in the DP-OM groups. Seven cases (53.8%) showed scattered fungal hyphae, and the other 3 cases had no fungal hyphae inside the nail plate. Serous lakes were more common in the DP-OM group than in the DP-TO group (38.5% vs. 8.7%: *P* = 0.073). No hemorrhage was detected in either the DP-OM or the DP-TO group.

## 4. Discussion

**Figure 1:**
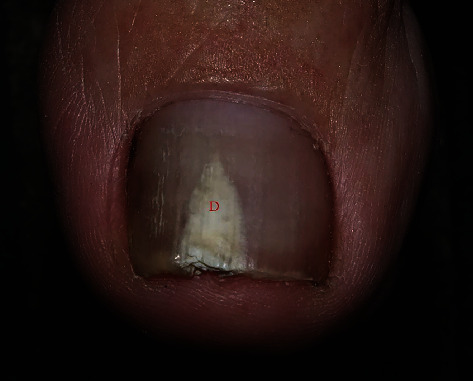
Dermatophytoma-like traumatic onychodystrophy. The figure shows central location (red “*D*”) and whitish homogenous color of dermatophytoma lesion.

**Figure 2:**
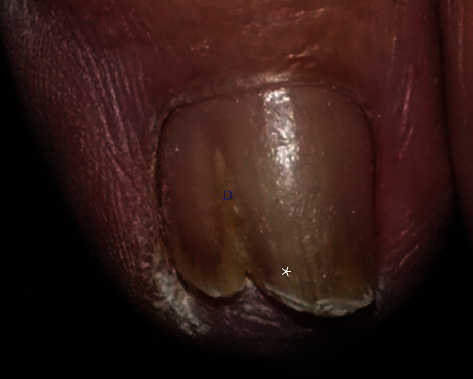
Dermatophytoma onychodystrophy. The figure shows the lateral location of the dermatophytoma (blue “*D*”) and longitudinal striae adjacent to the dermatophytoma (white asterisk).

The current investigation found clinical clues that will help to make diagnoses. DP-TO was associated with centrally located dermatophytoma lesions, homogenous white discoloration, and sharp-edged onycholysis (Figure 1). In contrast, DP-OM was related to a lateral location, adjacent striae, sulfur-nugget-like debris (yellow clumps), and ipsilateral foot scale (Figure 2).

The risk factors associated with OM were diabetes, peripheral vascular disease, psoriasis, advancing age, immunosuppressive therapy, agriculture-related occupation, living with family members who have OM, excessive perspiration, and pets [17, 18]. From this study, the demographic data and risk factors associated with OM might not be able to distinguish between DP-OM and DP-TO. This limitation may be because some risk factors were found with both nail disorders, such as being elderly and having a history of trauma.

A dermoscopic examination is a valuable tool for discerning between DP-OM and DP-TO. In the hyponychium view, a finding of yellow clumping debris in a ruin appearance, known as “sulfur nuggets,” is a prime indicator of DP-OM. Consistent with a previous study, the current investigation found that the presence of sulfur nuggets was significantly associated with OM [14].

Regarding the dorsal view of dermoscopy, this study found that the color of dermatophytoma-like lesions helps discriminate DP-OM from DP-TO. A homogenous, whitish lesion was significantly present in DP-TO. Unlike traumatic causes, the discoloration in DP-OM may be responsible for the change in the nail microbiome. Brown and black discoloration stemmed from melanin-producing organisms or melanin activation caused by inflammation of fungal invasion [19]. A case was also reported of a secondary Pseudomonas infection in OM causing green nails [20].

Another valuable clue for separating DP-OM and DP-TO is the location of their lesions. Those on the lateral side of the nail plate were associated with DP-OM. In contrast, approximately half of the DP-TO lesions were located centrally. The lateral location of DP-OM may be similar to lateral subungual hyperkeratosis of OM, which is caused by fungal invasion via the lateral side of a nail. The central location of DP-TO may be related to direct traumatic force to the nail center.

Moreover, longitudinal striae adjacent to dermatophytoma were more common in DP-OM. This finding is consistent with the previous study reported that longitudinal streak was commonly observed in onychomycosis [21]. The linear edge of the onycholytic area was found to be significantly associated with DP-TO. This feature had also been observed in traumatic onychodystrophy [6]. Thus, dermoscopic examination is a valuable means of distinguishing between DP-OM and DP-TO. Both hyponychium and dorsal views are required for a holistic examination. Additionally, a foot examination is vital for diagnosing fungal nail infections as fungal foot infections commonly occur with OM. The current work found that scales on the ipsilateral foot and ipsilateral tinea pedis were significantly associated with DP-OM [22–24].

As for the nail histopathology, there were few cases of DP-TO with parakeratosis solely on the upper half of the nail plate. The parakeratosis might have resulted from a direct force on the dorsal surface of the upper half of the nail plate. In contrast to DP-OM, some cases of DP-TO showed parakeratosis exclusively on the lower half of the nail plate. The parakeratosis was probably due to molds invading from the subungual side. There is scant literature on the histopathology of TO. The current investigation found that the features found in DP-TO were parakeratosis exclusively on the upper half of the nail plate, serous lakes, and a bacterial presence. Neutrophils were found only in the DP-OM group, which suggests that neutrophils are uncommon in DP-TO. These findings were consistent with the work of Neves and colleagues, who reported that only 2.9% of TO presented with neutrophils [25]. Moreover, hemorrhage may not always be observed in traumatic cases.

This study had a few limitations. First, the sample size was limited: only patients with complete mycological investigations and histopathology results from nail clipping were enrolled. Further studies with larger sample sizes may be needed. Second, the study design was retrospective; so, missing data and selection bias could not be avoided. Lastly, drilling technique was not used as a standard method for specimen collection in this study. Since specimen collection for DP-OM is not easily done, the drilling technique is preferred to increase the yield of KOH examination and fungal culture [26, 27].

## 5. Conclusions

Dermatophytoma lesions can be caused by fungal infections or trauma. It is crucial to establish a correct diagnosis since the causative conditions are treated differently. A foot examination and a dermoscopic inspection with dorsal and hyponychium views are essential to a definitive diagnosis. Sulfur-nugget-like debris, a lateral location of dermatophytoma, adjacent striae, scaly foot, and tinea pedis on the ipsilateral foot are significantly associated with DP-OM.

## Figures and Tables

**Table 1:** Comparison of nail, surrounding tissue, and foot features of dermatophytoma onychomycosis and dermatophytoma-like traumatic onychodystrophy.

Characteristic	Number (%)		
	Dermatophytoma onychomycosis (*n* = 13)	Dermatophytoma-like traumatic onychodystrophy (*n* = 23)	*P* value
Nail finding			
Nail thickness; mm (mean ± SD)	1.4 ± 0.9	1.4 ± 0.6	1.000
Depth of nail involvement			0.597
Less than 1/3	2 (15.4)	3 (13.0)	
1/3–2/3	9 (69.2)	19 (82.6)	
>2/3	2 (15.4)	1 (4.3)	
Location of dermatophytoma lesions			0.008^∗^
Center	1 (7.7)	12 (52.2)	
Lateral	12 (92.3)	11 (47.8)	
Splinter hemorrhage	0 (0)	2 (8.7)	0.525
Discoloration	13 (100)	23 (100)	—
Yellow	6 (46.2)	5 (21.7)	0.127
Brown to black	2 (15.4)	8 (34.8)	0.212
White	11 (84.6)	21 (91.3)	0.609
Green	2 (15.4)	1 (4.3)	0.250
Surrounding nail tissue			
Toe tip callus	0 (0)	4 (17.4)	0.274
Onychophosis	6 (46.2)	11 (47.8)	0.923
Ipsilateral foot with dermatophytoma nail lesions			
Foot deformity	5 (38.5)	5 (21.7)	0.282
Hallux vulgus	4 (30.8)	4 (17.4)	0.354
Claw toe	1 (7.7)	1 (4.3)	1.000
Overriding toe	0 (0)	3 (13.0)	0.288
Scale	9 (69.2)	2 (8.7)	<0.001^∗^
Callus	2 (15.4)	2 (8.7)	0.609

Abbreviation: SD: standard deviation.

**Table 2:** Comparison of dermoscopic nail findings of dermatophytoma and dermatophytoma-like traumatic onychodystrophy.

Characteristic	Number (%)		
	Dermatophytoma onychomycosis (*n* = 13)	Dermatophytoma-like-traumatic onychodystrophy (*n* = 23)	*P* value
Dorsal view			
(i) Homogenous whitish discoloration	1 (7.7)	11 (47.8)	0.014^∗^
(ii) Heterogenous multicoloration	5 (38.5)	5 (21.7)	0.282
(iii) Sharp edge of onycholytic area	0 (0)	10 (43.5)	0.005^∗^
(iv) Longitudinal striae adjacent to dermatophytoma	9 (69.2)	7 (30.4)	0.024^∗^
(v) Spiked jagged proximal edge	9 (69.2)	10 (43.5)	0.177
(vi) Distal nicking	1 (7.7)	6 (26.1)	0.382
Hyponychium view			
(i) Sulfur-nugget-like subungual debris	3 (23.1)	0 (0)	0.040^∗^
(ii) “Ruin appearance”	7 (53.8)	11 (47.8)	0.729
(iii) Onychauxis	4 (30.8)	11 (47.8)	0.319

**Table 3:** Comparison of histopathology of nail of dermatophytoma and dermatophytoma-like onychodystrophy.

Characteristic	Number (%)		
	Dermatophytoma onychomycosis (*n* = 13)	Dermatophytoma-like traumatic nail dystrophy (*n* = 23)	*P* value
Parakeratosis	11 (84.6)	19 (82.6)	0.124
Location of parakeratosis			0.360
Upper	0 (0)	2 (8.7)	
Lower (subungual)	2 (15.4)	1 (4.3)	
Both upper and lower	11 (84.6)	20 (87.0)	
Hemorrhage	0 (0)	0 (0)	—
Serous lake	5 (38.5)	2 (8.7)	0.073
Neutrophil infiltration	1 (7.7)	0 (0)	0.361
Presence of bacteria	9 (69.2)	17 (73.9)	0.678
Fungal hyphae invading inside nail plate			
No	3 (23.1)	23 (100.0)	<0.001^∗^
Scatter	7 (53.8)	0 (0)	
Clumping fungal hyphae	3 (23.1)	0 (0)	

## Data Availability

Data in this study are available upon reasonable request. Please contact the corresponding author for the data request.

## References

[1] Burkhart C. N., Burkhart C. G., Gupta A. K. (2002). Dermatophytoma: recalcitrance to treatment because of existence of fungal biofilm. *Journal of the American Academy of Dermatology*.

[2] Martinez-Herrera E., Moreno-Coutiño G., Fernández-Martínez R. F., Finch J., Arenas R. (2012). Dermatophytoma: description of 7 cases. *Journal of the American Academy of Dermatology*.

[3] Sigurgeirsson B. (2010). Prognostic factors for cure following treatment of onychomycosis. *Journal of the European Academy of Dermatology and Venereology*.

[4] Martínez-Herrera E. O., Arroyo-Camarena S., Tejada-García D. L., Porras-López C. F., Arenas R. (2015). Onychomycosis due to opportunistic molds. *Anais Brasileiros de Dermatologia*.

[5] Leeyaphan C., Bunyaratavej S., Prasertworanun N., Muanprasart C., Matthapan L., Rujitharanawong C. (2016). Dermatophytoma: an under-recognized condition. *Indian Journal of Dermatology, Venereology and Leprology*.

[6] Ramos Pinheiro R., Dias Domingues T., Sousa V., Galhardas C., Apetato M., Lencastre A. (2019). A comparative study of onychomycosis and traumatic toenail onychodystrophy dermoscopic patterns. *Journal of the European Academy of Dermatology and Venereology*.

[7] Gupta A. K., Stec N., Summerbell R. C. (2020). Onychomycosis: a review. *Journal of the European Academy of Dermatology and Venereology*.

[8] Tucker J. R. (2015). Nail deformities and injuries. *Primary Care*.

[9] Zaias N., Rebell G., Escovar S. (2014). Asymmetric gait nail unit syndrome: the most common worldwide toenail abnormality and onychomycosis. *Skinmed*.

[10] Weinberg J. M., Koestenblatt E. K., Tutrone W. D., Tishler H. R., Najarian L. (2003). Comparison of diagnostic methods in the evaluation of onychomycosis. *Journal of the American Academy of Dermatology*.

[11] Mishlab S., Avitan-Hersh E., Bergman R. (2021). Histopathological findings in nail clippings with periodic acid-Schiff-positive fungi. *The American Journal of Dermatopathology*.

[12] Gupta A. K., Mays R. R., Versteeg S. G., Shear N. H., Piguet V. (2018). Update on current approaches to diagnosis and treatment of onychomycosis. *Expert Review of Anti-Infective Therapy*.

[13] Kayarkatte M. N., Singal A., Pandhi D., Das S., Sharma S. (2020). Nail dermoscopy (onychoscopy) findings in the diagnosis of primary onychomycosis: a cross-sectional study. *Indian Journal of Dermatology, Venereology and Leprology*.

[14] Leeyaphan C., Suphatsathienkul P., Limphoka P., Kiratiwongwan R., Bunyaratavej S. (2021). Sulphur Nuggets. *Medical mycology journal*.

[15] Piraccini B. M., Balestri R., Starace M., Rech G. (2013). Nail digital dermoscopy (onychoscopy) in the diagnosis of onychomycosis. *Journal of the European Academy of Dermatology and Venereology*.

[16] Bennett D., Rubin A. I. (2013). Dermatophytoma: a clinicopathologic entity important for dermatologists and dermatopathologists to identify. *International Journal of Dermatology*.

[17] Gupta A. K., Versteeg S. G., Shear N. H. (2017). Onychomycosis in the 21st century: an update on diagnosis, epidemiology, and treatment. *Journal of Cutaneous Medicine and Surgery*.

[18] Leeyaphan C., Bunyarata S., Chadchavalpanichaya N., Rujitharanawong C., Phaitoonwattanakij S., Matthapan L. (2018). Clinical and laboratory findings in trauma-induced nail dystrophy versus onychomycosis. *Siriraj Medical Journal*.

[19] Finch J., Arenas R., Baran R. (2012). Fungal melanonychia. *Journal of the American Academy of Dermatology*.

[20] Elewski B. E. (1997). Bacterial infection in a patient with onychomycosis. *Journal of the American Academy of Dermatology*.

[21] Yorulmaz A., Yalcin B. (2018). Dermoscopy as a first step in the diagnosis of onychomycosis. *Advances in Dermatology and Allergology*.

[22] Bunyaratavej S., Limphoka P., Kiratiwongwan R., Leeyaphan C. (2020). Eclipsed phenomenon: the relationship between nail and foot infections in patients presenting with nondermatophyte infections after dermatophyte infections in onychomycosis. *The British Journal of Dermatology*.

[23] Szepietowski J. C., Reich A., Garlowska E., Kulig M., Baran E., Onychomycosis Epidemiology Study Group (2006). Factors influencing coexistence of toenail onychomycosis with tinea pedis and other dermatomycoses: a survey of 2761 patients. *Archives of Dermatology*.

[24] Daniel C. R., Jellinek N. J. (2006). The pedal fungus reservoir. *Archives of Dermatology*.

[25] Neves J. M., Cunha N., João A., Lencastre A. (2019). Neutrophils in nail clipping histology: a retrospective review of 112 cases. *Skin Appendage Disorders*.

[26] Shemer A., Gupta A. K., Amichai B. (2016). An open comparative study of nail drilling as adjunctive treatment for toenail onychomycosis (.). *The Journal of Dermatological Treatment*.

[27] Shemer A., Trau H., Davidovici B., Grunwald M. H., Amichai B. (2008). Collection of fungi samples from nails: comparative study of curettage and drilling techniques. *Journal of the European Academy of Dermatology and Venereology*.

