# Corrigendum to “Clinical Clues to Differentiate Between Dermatophyte Onychomycosis (DP-OM) and Dermatophytoma-Like Traumatic Onychodystrophy (DP-TO)”

**DOI:** 10.1155/bmri/9862803

**Published:** 2025-07-28

S. Bunyaratavej, P. Pattanaprichakul, P. Sitthinamsuwan, et al., “Clinical Clues to Differentiate Between Dermatophyte Onychomycosis (DP-OM) and Dermatophytoma-Like Traumatic Onychodystrophy (DP-TO),” *BioMed Research International* 2022 (2022), https://doi.org/10.1155/2022/8519376

The ethics approval number stated in the article is incorrect; the correct ethics approval number is SI 724/2020.

The authors apologize for this error.

